# Modeling contributions of cognition and apathy to functional impairment in younger‐onset dementia

**DOI:** 10.1002/dad2.70249

**Published:** 2026-01-20

**Authors:** Samuel L. Warren, Rebekah M. Ahmed, Olivier Piguet, David Foxe, Muireann Irish

**Affiliations:** ^1^ The University of Sydney Brain & Mind Centre Camperdown NSW Australia; ^2^ The University of Sydney School of Psychology Camperdown NSW Australia; ^3^ Memory and Cognition Clinic Institute of Clinical Neurosciences Royal Prince Alfred Hospital Sydney NSW Australia

**Keywords:** Alzheimer's disease, apathy, cognition, frontotemporal dementia, functional impairment, generalized additive model, younger‐onset dementia

## Abstract

**INTRODUCTION:**

Overlaps in symptom presentation limits the capacity to predict functional impairment and future care needs in younger‐onset dementia syndromes.

**METHODS:**

A general additive model (GAM) was applied to cross‐sectional retrospective data from 375 participants with younger‐onset dementia; 152 behavioral‐variant frontotemporal dementia (bvFTD), 118 Alzheimer's disease (AD), 66 semantic dementia, and 39 progressive nonfluent aphasia (PNFA). This GAM aimed to explore the dynamic interrelationships between established measures of global cognition, apathy, and functional impairment.

**RESULTS:**

Our GAM significantly predicted functional impairment in all syndromes with a high explained variance (59.5%). Cognition and apathy emerged as significant predictors of functional impairment in each syndrome (*p*‐values < .015). These relationships were consistently linear in AD, non‐linear in SD, and mixed in bvFTD and PNFA (i.e., cognition linear and apathy non‐linear).

**DISCUSSION:**

Our study shows the potential prognostic utility of GAMs for identifying syndrome‐specific transition periods across group‐level staging's of functional impairment.

**Highlights:**

- First study to apply a general additive model to functional impairment in younger‐onset dementia.
- Studied 375 individuals with younger‐onset Alzheimer's disease or frontotemporal dementia.
- Apathy and cognition were significant predictors of functional impairment in all syndromes.
- This modeling has significant implications for syndrome‐specific prognosis and management.

## 1 BACKGROUND

Mounting evidence reveals considerable overlap in symptom presentation across younger‐onset dementia syndromes, including frontotemporal dementia (FTD) and Alzheimer's disease (AD).^1^, ^2^ It has been suggested that advancing disease severity may drive within‐group heterogeneity resulting in overlapping symptom profiles.^3^ Such symptomatic variability is problematic not only for conferring an accurate diagnosis but in developing robust prognostic indicators to inform treatment options and care.^4^, ^5^ Group studies modeling symptom trajectories in younger‐onset dementia suggest that overall cognition can be used to predict functional impairment in FTD and AD. These studies, however, are based on the assumption that cognition and functional outcomes decline in a predictable and linear fashion over time.^6^, ^7^, ^8^, ^9^, ^10^ In practice, this linear assumption does not generalize to all younger‐onset dementia populations, such as preclinical AD.^11^, ^12^, ^13^ Moreover, most studies to date have limited the focus to cross‐sectional pairwise comparisons between syndromes (e.g., behavioral variant FTD [bvFTD] vs. AD).^14^, ^15^, ^16^, ^17^ It therefore remains unclear how cognitive and behavioral profiles coalesce across younger‐onset dementia syndromes and their respective utility in predicting functional outcomes.^8^

While cognition has tended to take center stage in predicting functional impairment in younger‐onset dementia, neuropsychiatric symptoms are increasingly understood to play a crucial role.^18^ Apathy (i.e., loss of goal‐directed, motivated behavior) is now well‐established as a transdiagnostic feature in younger‐onset dementia.^3^, ^19^, ^20^, ^21^, ^22^ This loss of motivation has been shown to differentially contribute to functional impairment across younger‐onset dementia syndromes,^23^, ^24^, ^25^ suggesting the need to integrate motivational changes into models of disease progression. Importantly, however, many neuropsychiatric symptoms exhibit a non‐linear trajectory over the disease course in younger‐onset dementia,^7^, ^26^ cautioning against the use of linear approaches when predicting disease outcomes. Notably, apathy unfolds in a non‐linear manner across some genetic variants of behavioral variant FTD (bvFTD) (i.e., *C9orf72* and *GRN*), increasing in severity early in the disease course, before reaching a plateau after 6–8 years.^6^ By relying exclusively on linear approaches to model disease outcomes in younger‐onset dementia, we risk overlooking important fluctuations with direct bearing on everyday function.

Generalized additive models (GAMs) are of immense interest in this context as they permit statistical modeling of overlapping and non‐linear data.^27^ Such approaches extend beyond linear predictions by using splines to approximate curves using multiple stacked functions.^28^ Through this hybrid parametric and non‐parametric approach, GAMs can perform the same statistical tasks as regression models (e.g., linear mixed effect, multiple regression, logistic regression) while also mapping non‐linear fluctuations in disease symptomatology. For example, GAMs have been used to predict and plot the nonlinear relationship between memory tests and functional impairment in AD.^13^ GAMs are therefore clinically advantageous as they use symptomatic profiles to distinguish dementia subtypes with overlapping features, and can help predict disease outcomes.

The objective of this study was to establish the utility of GAMs for capturing the inherent heterogeneity and overlap in symptomatology across younger‐onset dementia syndromes, and to mirror the diversity of symptom presentations typically seen in clinical practice. Specifically, we sought to (i) account for overlapping clinical features across younger‐onset dementia subtypes and (ii) model the natural unfolding of specific features, namely cognition and motivation, as predictors of functional impairment. We selected global cognition as it has been shown to reliably predict functional impairment in AD^16^, ^29^ and is an optimal first step for applying GAMs to younger‐onset dementia. Similarly, apathy was chosen as it is the most common non‐cognitive symptom in dementia and particularly relevant for functional outcomes in FTD.^24^, ^25^ Crucially, we predicted that cognition would follow a linear predictive trajectory in AD where lower scores predict poorer functional ability. In contrast, we hypothesized that apathy would present in a non‐linear fashion in FTD, predicting poorer functional ability irrespective of FTD clinical phenotype. In doing so, we aimed to highlight the utility of GAMs for cross‐sectional staging of group‐level symptomatic trajectories on a functional disease timeline.

## 2 METHODS

### 2.1 Participants

A retrospective sample of 375 participants diagnosed with younger‐onset dementia was included, recruited between November 2007 and February 2024 through FRONTIER, the frontotemporal dementia research clinic at the Brain and Mind Centre, University of Sydney, Australia. This cross‐sectional sample consisted of 152 bvFTD, 118 AD, 66 semantic dementia (SD), and 39 progressive nonfluent aphasia (PNFA) individuals at their first clinical visit. All participants underwent comprehensive clinical, neurological, and neuropsychological assessment along with structural neuroimaging (i.e., magnetic resonance imaging [MRI]). Clinical diagnoses proceeded in line with current diagnostic criteria for clinically probable bvFTD,^30^ primary progressive aphasia,^31^ and AD.^32^ Atypical language (i.e., logopenic progressive aphasia) and visual (i.e., posterior cortical atrophy) variants of AD were not included in the AD group. For full details of clinical and neuropsychological testing, see Foxe et al.^8^, ^33^

**RESEARCH IN CONTEXT**

1. **Systematic review**: We reviewed the literature on functional impairment in younger‐onset dementia (YOD) and non‐linear modeling using common databases (e.g., Google Scholar and PubMed). While some studies had used linear and curvilinear modeling in YOD, we found no studies that predicted functional impairment across multiple YOD syndromes using general additive models (GAMs). Existing literature, and gaps therein, are discussed throughout.
2. **Interpretation**: Our findings reveal distinct contributions of cognition and apathy to functional outcomes in YOD. This work enables more accurate determination of sensitive symptomatic periods at the group level, from which proactive care planning can be implemented.
3. **Future directions**: GAMs offer significant utility for mapping complex relationships between symptoms and functional outcomes in YOD. Future studies could extend this approach to other forms of YOD (e.g., vascular dementia, dementia with Lewy bodies), and to examine temporal dynamics of symptom progression (e.g., event‐based modeling) in these syndromes.

### 2.2 Measures

Functional impairment was indexed using the Frontotemporal Dementia Rating Scale (FRS),^34^ a 30‐item career report questionnaire probing functional ability across several domains (i.e., behaviors, outings and shopping, household chores and telephone, finances, medications, meal preparation and eating, and self‐care and mobility) in terms of frequency of symptoms (i.e., all the time, sometimes, or never). Scores are subjected to a Rasch analysis, which converts the FRS categorical answers to a logit disease severity score ranging from 5.39 to −6.66, where lower values reflect greater functional impairment.^34^ While initially made for FTD populations, the FRS has been shown to reliably index functional impairment in AD and is a useful tool when attempting to determine functional impairment across different dementia subtypes.^35^

Cognition was measured using the Addenbrooke's Cognitive Examination III (ACE‐III)^36^ a general cognitive screening instrument that measures the domains of memory, attention, language, fluency, and visuospatial ability. The ACE‐III total score ranges from 0–100, with lower scores denoting worse cognitive ability. A subset of participants (*n* = 44) tested prior to 2013 completed the older ACE‐R, and scores were converted to corresponding ACE‐III scores using established algorithms.^37^

Apathy was measured using the motivation subscale of the Cambridge Behavioural Inventory Revised (CBI‐R),^38^ which comprises five items probing the frequency of carer‐rated motivational disturbances experienced by the patient over the last month. Scores range from 0 to 20, where higher scores indicate greater motivational disturbances (i.e., apathy). To avoid confusion between the construct and scale, we refer to the scale as CBI‐Apathy from here on.

### 2.3 Statistical analyses

Descriptive statistics were performed using IBM SPSS statistics version 29. Prior to analyses, distributions for all continuous variables or residuals were checked using Shapiro Wilk tests. Group comparisons were performed using a Kruskal‐Wallis Test with pairwise contrasts and Bonferroni corrections due to non‐normally distributed residuals (e.g., age, apathy, cognition). Categorical variables (e.g., sex) were compared using Chi‐squared tests. Approximately 6.6% of the CBI‐R and 11.5% of FRS data were missing. To ensure a complete sample, missing data were imputed using a custom script using Multivariate Imputation by Chained Equations (MICE)^39^ in Python version 3.12.5 and the iterative imputer function from scikit‐learn version 1.5.2. All variables of interest were used to impute missing values using a Bayesian ridge method with 1000 iterations.

A GAM was run in R version 4.4.1 and version 1.9‐1 of the mgcv library.^27^ The FRS logit score was set as the dependent variable, while the ACE‐III total and CBI‐Apathy scores were independent variables, and age and education were included as covariates. Disease duration was not included as a covariate due to its high concurvity/collinearity with other variables (e.g., age). The independent variables were split by diagnosis using an interaction effect to facilitate group comparisons and predictions. The restricted maximum likelihood (REML) method was used to estimate smoothing parameters, and k (the maximum number of splines) was set to default for all variables except for CBI‐Apathy (*k* = 4) and ACE‐III (*k* = 5). These decreases in splines were fine‐tuned using the k significance testing in mgcv's gam check function to avoid violations of concurvity.^27^ Group sizes were not balanced, however, GAMs are robust to class imbalances and we wanted to maximize statistical power.^28^

The initial GAM violated the assumptions of homoscedasticity and normality of the residuals. These violations were addressed using a scaled *t*‐family GAM with a log function. FRS logit scores were increased by 3.09 (i.e., the lowest negative value) so that all values were positive, and a log transformation was possible.

## 3 RESULTS

### 3.1 Descriptive statistics

Table 1 presents the descriptive statistics for demographic, independent variables, model covariates, and associated post‐hoc testing. A chi‐squared test displayed a significant difference in sex distributions between diagnostic groups (*p* = .006). Kruskal–Wallis tests revealed no significant differences across the patient groups in terms of years in education, and disease duration (years elapsed since symptom onset). A significant group difference was found for age, reflecting the younger age in bvFTD compared with AD and PNFA; however, this comparison did not survive Bonferroni correction for multiple comparisons. The severity of cognitive dysfunction on the ACE‐III also differed across the groups, driven by significantly poorer performance in AD and SD compared to the bvFTD and PNFA groups. Finally, carer‐rated apathy on the CBI‐R was significantly higher in bvFTD relative to all other groups. No other group differences were evident (all *p* values > .097).

**TABLE 1:** Demographic, clinical, and cognitive characteristics of the study sample

Variable	AD *n* = 118	bvFTD *n* = 152	PNFA *n* = 39	SD *n* = 66	Test statistic	*p‐*value	Post‐hoc comparison
Sex [% Female]	47.5%	30.9%	53.8%	48.5%	χ = 12.50	0.006	–
Age [years]	66.1(8.6)	63.1(8.4)	67.8(9.7)	65.2(7.1)	*H* = 9.52	0.023	^a^bvFTD < AD & PNFA
Education [years]	12.2(3.1)	12.6(3.0)	12.4(3.1)	12.5(3.1)	*H* = 0.27	0.966	–
Disease duration [years]	4.3(2.3)	5.2(3.5)	4.0(1.9)	5.1(2.6)	*H* = 6.33	0.097	–
ACE‐III Total [100]	63.1(19.3)	76.5(17.1)	74.9(15.4)	59.1(18.2)	*H* = 67.27	<.001	SD & AD < bvFTD & PNFA
CBI‐R Apathy [20]	6.0(5.0)	11.5(5.8)	4.5(5.2)	7.2(6.1)	*H* = 75.01	<.001	other < bvFTD

Note: Values represent means with standard deviation in parentheses. Maximum test score and/or unit of measurement provided in square brackets where appropriate.

Abbreviation: ACE‐III, Addenbrooke's Cognitive Examination III; AD, Alzheimer's disease; bvFTD, behavioral variant frontotemporal dementia; CBI‐R, Cambridge Behavioural Inventory Revised; *H*, Kruskal–Wallis; PNFA, progressive nonfluent aphasia; SD, semantic dementia; *χ*, chi‐squared.

^a^ Not significant following Bonferroni correction for multiple comparisons.

### 3.2 Predicting functional impairment using global cognition

**FIGURE 1:**
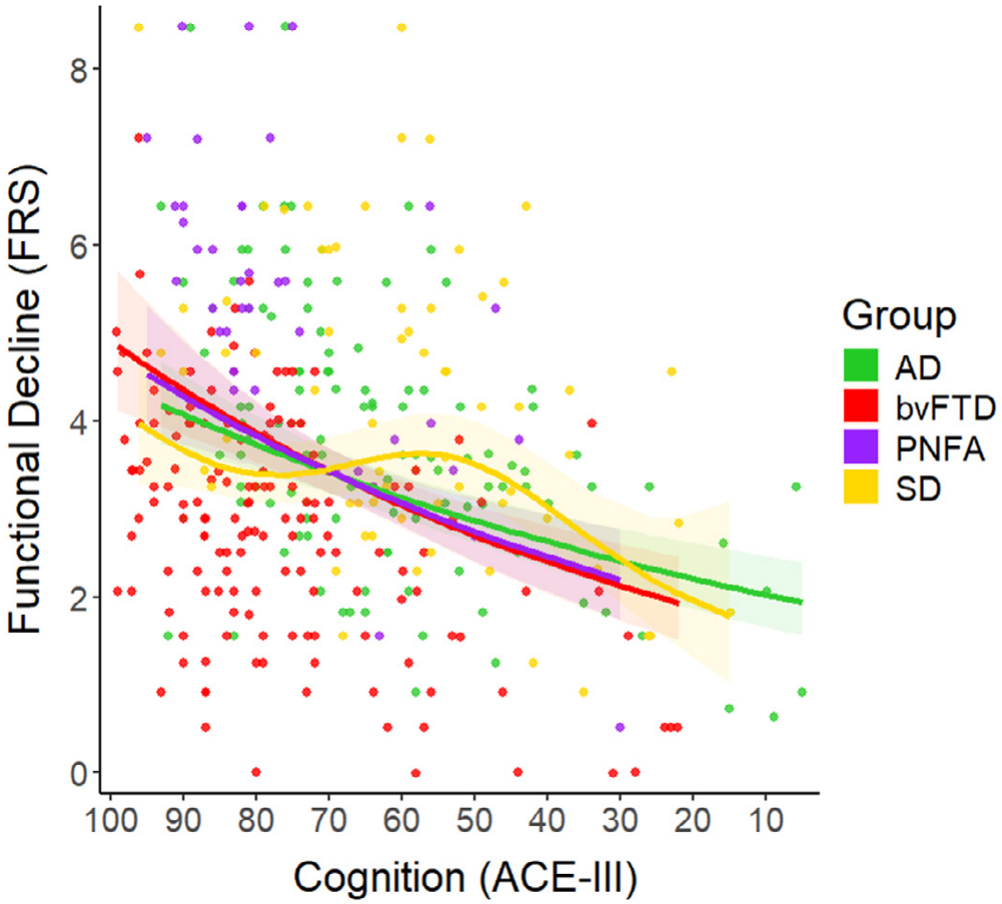
GAM predicting functional impairment using cognition in younger‐onset dementia. Note: Functional impairment was measured using the FRS while cognition was assessed using the ACE‐III. For both tests, lower values denote poorer performance. The FRS was transformed so that all values were positive to facilitate a scaled t‐family GAM with a log function (see statistical analysis section for further details). ACE‐III, Addenbrooke's Cognitive Examination III; AD, Alzheimer's disease, bvFTD, behavioral‐variant frontotemporal dementia; FRS, Frontotemporal Dementia Rating Scale; GAM, generalized additive model; PNFA, progressive nonfluent aphasia; SD, semantic dementia

The GAM converged in nine iterations and achieved a REML score of 590.73 and a strong accounted variance of 59.5% (adjusted *r* ^2^ = .595, deviance explained = 55.8%). Table 2 displays the GAM results from each predictor of functional impairment in each group. Cognitive function (i.e., ACE‐III Total) significantly predicted functional impairment in each dementia subtype. Notably, these cognitive predictions were mostly linear, except for in SD. These effects were indicated by the effective degrees of freedom (edf), where a score of one indicates linearity and higher scores denote increasing non‐linearity. The predictive curves mapping the relationship between ACE‐III total scores and functional impairment were relatively comparable across AD, PNFA, and bvFTD groups, with variations at the extremities of ACE‐III total scores (Figure 1). In contrast, the cognitive prediction in the SD group showed moderate‐strong non‐linearity (edf = 2.92), with functional impairment remaining relatively stable up until participants reached an ACE‐III total score of 55, following which functional impairment decreased sharply.

**TABLE 2:** GAMM predictors of functional impairment by diagnostic group

Domain	Predictors	edf	Ref. df	Chi squared	*p*‐value
Demographic covariates	Age	1.00	1.00	0.12	0.727
	Education	1.00	1.00	1.97	0.161
Cognition (ACE‐III)	AD	1.00	1.00	28.18	<.001^a^
	bvFTD	1.00	1.00	23.18	<.001^a^
	PNFA	1.00	1.00	15.37	<.001^a^
	SD	2.92	3.37	10.66	0.015^a^
Apathy (CBI‐Apathy)	AD	1.00	1.00	50.50	<.001^a^
	bvFTD	1.67	2.03	37.76	<.001^a^
	PNFA	1.86	2.19	19.57	<.001^a^
	SD	2.12	2.51	66.11	<.001^a^

*Note*: Apathy was measured using the Cambridge Behavioural Inventory Revised (CBI‐R) motivation subscale, cognition using the Addenbrooke's Cognitive Examination III (ACE‐III) total score, and functional impairment using the Frontotemporal Dementia Rating Scale (FRS) logit score.

Abbreviation: AD, Alzheimer's disease; bvFTD, behavioral‐variant frontotemporal dementia; edf, effective degrees of freedom; PNFA, progressive nonfluent aphasia; Ref. df, reference degrees of freedom; SD, semantic dementia.

^a^ Significant at *α* = .05 level.

### 3.3 Apathy as a predictor of functional impairment

**FIGURE 2:**
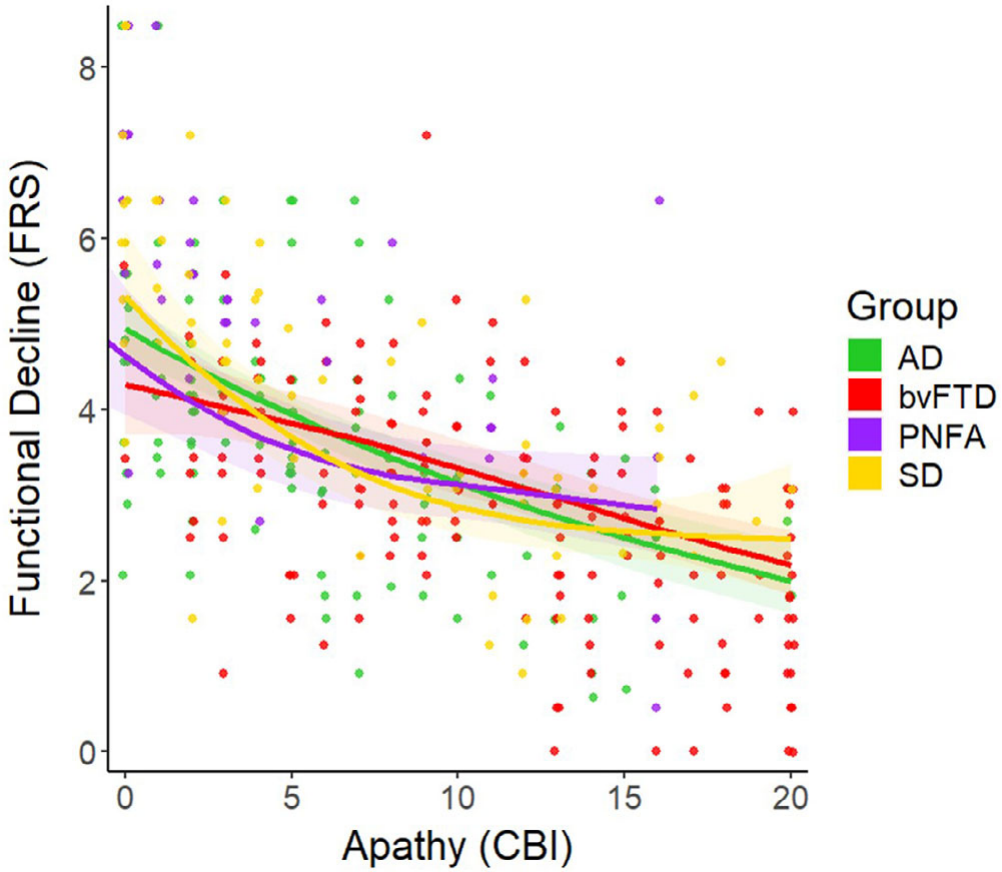
GAM predicting functional impairment using apathy in younger‐onset dementia. Note: Functional impairment was measured using the FRS where lower FRS values denote worsening functional impairment. Apathy was measured using the motivation subscale of the CBI‐R, where higher values denote greater apathy severity. The FRS was transformed so that all values were positive to facilitate a scaled *t*‐family GAM with a log function (see statistical analysis section for further details). AD, Alzheimer's disease; bvFTD, behavioral‐variant frontotemporal dementia; CBI‐R, Cambridge Behavioural Inventory Revised; FRS, Frontotemporal Dementia Rating Scale; GAM, generalized additive model; PNFA, progressive nonfluent aphasia; SD, semantic dementia

Apathy (i.e., CBI‐Apathy) emerged as a significant predictor of functional impairment across all younger‐onset dementia subtypes (Table 2). Out of these predictors, only the AD group had a linear prediction (edf = 1.00), with nonlinearity greatest in SD (edf = 2.12), followed by PNFA (edf = 1.86) and then bvFTD (edf = 1.67). The GAM indicated that as levels of apathy increase, bvFTD and AD groups showed the most severe level of functional impairment. In contrast, SD and PNFA groups showed a steeper gradient of functional impairment with apathetic symptoms early on until a midpoint (CBI‐apathy ∼5–10), beyond which functional impairment appeared to stabilize despite increasing levels of apathy. Figure 2 illustrates these trajectories of apathy for each younger‐onset dementia group.

### 3.4 Differences in sex and functional impairment

Parametric group comparisons on the FRS were run as part of the GAM analysis. Results indicated that bvFTD group showed greater functional impairment relative to AD while SD and PNFA showed milder functional impairments (Table S1). We re‐ran the GAM with sex as an additional covariate; however, sex was non‐significant and the overall results remained unchanged (Tables S2 & S3).

## 4 DISCUSSION

Younger‐onset dementia syndromes exhibit complex and often overlapping changes in cognition and behavior. How such changes relate to functional outcomes, however, remains unclear. This study used a GAM to predict functional impairment in several younger‐onset dementia syndromes (bvFTD, SD, PNFA, AD) using cognition and apathy as predictors. Overall, we found distinct group‐level trajectories of cognitive impairment and apathy for all younger‐onset dementia syndromes studied; however, the nature of these predictions differed by dementia subtype across the scale of functional impairment. Irrespective of outcome measure, linear relationships were observed for younger‐onset AD while non‐linear relationships were consistently found for SD. Meanwhile, bvFTD and PNFA subtypes showed a mixture of linear (cognition) and non‐linear (apathy) profiles. Our study addresses clinician‐led calls for non‐linear methods that can capture complex group‐level trends in clinical profiles^40^ and highlights the utility of GAMs for capturing distinct symptomatic relationships in younger‐onset dementia syndromes.

Considering cognition first, we found that global performance on the ACE‐III significantly predicted functional impairment irrespective of dementia subtype. This finding is in keeping with an established literature relating cognitive dysfunction to functional outcomes in younger‐onset dementia^8^, ^10^ and corroborates the interplay between loss of cognitive function and difficulties in performing daily activities.^33^ Notably, we found linear relationships between cognition and functional impairment in younger‐onset AD, bvFTD, and PNFA, whereby lower ACE‐III total scores predicted poorer function. Crucially, however, the SD group did not show this linear relationship between cognitive impairment and functional impairment. Rather, a nonlinear relationship was observed where functional impairment in SD remained relatively stable, until a critical tipping point of cognitive dysfunction on the ACE‐III (score = 55/100). Beyond this cognitive threshold, functional abilities in SD diminished sharply, observable as a step‐change from “moderate” to “severe” functional impairment on the FRS. Our findings caution against a “one‐size‐fits‐all” approach when using cognitive cutoffs to inform functional impairment in younger‐onset dementia syndromes and suggest that different cognitive thresholds may hold utility in syndrome‐specific adaptive care planning.

Our findings of a non‐linear relationship between cognitive dysfunction and functional impairment in SD go some way towards reconciling disparities in the literature, given that previous studies have failed to show a significant relationship between cognition and function in this syndrome.^37^, ^41^ Importantly, while the cognitive profile of SD is dominated by semantic degradation, several cognitive domains remain relatively preserved, including attention, visuospatial, and non‐conceptually loaded forms of episodic memory.^42^, ^43^ SD patients often remain oriented in space and time, can navigate within familiar environments, and display intact forms of prospective memory.^44^ We speculate that these islands of preservation may scaffold everyday functions (e.g., self‐care) much more strongly than language capacity.^45^ That is, preserved attention, orientation, spatial memory, and episodic memory may be more important predictors of functional outcomes in SD than indices of language or semantic processing, enabling the individual to maintain aspects of independence and self‐care. This is supported by findings on the stability of functional decline in the first five years of SD^23^, and in part, by the observed trajectories of these cognitive domains longitudinally.^33^, ^46^ Clarifying whether discrete domains of cognition scaffold functional outcomes at different stages of disease in SD represents a crucial avenue for future studies. Moreover, the potential mitigating role of cognitive reserve in supporting functional outcomes in SD, requires further focused investigation.

Interestingly, apathy emerged as a significant non‐linear predictor of functional impairment in all younger‐onset dementia syndromes except for AD, where a linear relationship was present. Our finding resonates with several studies demonstrating that functional impairment in FTD is dramatically impacted by loss of motivation.^20^, ^25^, ^47^ While previous studies have reported non‐linear profiles of apathy in bvFTD,^6^, ^7^ we show here how this non‐linear relationship impacts functional outcomes across a range of younger‐onset dementia syndromes within a singular model. Notably, functional impairment was tightly coupled with apathy early in the disease course of SD and PNFA, yet plateaued at a “moderate” level of severity on the FRS once a critical threshold was reached (CBI‐Apathy > 10). For younger‐onset AD, apathy and functional impairment exhibited a linear relationship, evident in a parallel and gradual progression of symptoms. Opposite to the other forms of FTD, apathy and functional impairment were relatively decoupled in the early course of bvFTD (CBI‐Apathy < 5) before exhibiting a gradual linear relationship similar to AD. These results highlight the deleterious impact of apathy on disease outcomes^48^ and underscores the need to consider loss of motivation when designing care plans to maintain functional ability in FTD.^49^

Our findings hold several important clinical implications. First, using the flexibility of GAMs, we successfully captured dynamic changes in cognition, apathy, and functional impairment within the same model, enabling us to not only study predictive relationships of specific symptoms, but to model group‐level differences in disease trajectories in reference to a functional disease timeline across younger‐onset dementia syndromes. This modeling approach may aid in the monitoring of functional impairment as an outcome measure in clinical trials, offering a more sensitive milestone of likely treatment response.^50^ Second, GAMs move us closer towards the clinical reality of neurodegenerative disorders where multiple features coalesce and synergistically impact the everyday independence of the individual. Of note is our finding that changes in cognition and motivation do not influence functional outcomes in the same manner across younger‐onset dementia syndromes, cautioning against a one‐size‐fits‐all approach in terms of symptom management and adaptive care planning. This inherent complexity speaks to the persistent but variable effect of cognitive and motivational disturbances on everyday function. Accordingly, the need to educate carers to identify individual‐level changes in these symptoms is paramount, enabling them to anticipate and respond to the changing symptom trajectory of the individual with dementia. This information has potential prognostic value enabling clinicians to identify critical windows for targeted intervention specific to each clinical subtype, which ultimately could ensure more proactive and tailored support is provided to patients and their families based on disease‐level symptom trajectories and downstream outcomes.

While our findings hold potential clinical utility, it is important to note that GAMs are limited by the same assumptions, statistical power, and group comparisons as linear models. These limitations mean that a GAM can only provide insights at the group level and cannot predict an individual's symptom trajectory or functional outcomes. Given these constraints, some caution is warranted before we can implement such approaches within clinical settings. For example, within our specific GAM, we note that our confidence intervals were wide, and splines restricted (i.e., k) due to splitting predictive variables by multiple diagnostic groups. As the first study to explore the utility of GAMs in younger‐onset dementia populations, our methods were constrained by a trade‐off between covering multiple domains (e.g., diseases, variables, covariates) and maintaining the stability of the model (e.g., concurvity and interpretability). As such, we constrained our focus to cognition and apathy, broadly defined, and admittedly coarsely measured.

Having confirmed the utility of GAMs in younger‐onset dementia, our findings provide a principled foundation for further studies to probe cognitive and neuropsychiatric symptomology in more detail. For example, it will be important to explore discrete domains of cognitive function (e.g., executive function, language, memory) in more detail, as well as distinguishing between key dimensions of apathy (e.g., affective, behavioral, cognitive) and anhedonia.^22^, ^25^ Further, while our covariate analyses did not suggest a significant effect of sex on our models, it will be important to tease apart the effect of demographic, socioeconomic and cultural factors on symptom expression and their relation to functional impairment in these syndromes. A larger independent sample incorporating pathological and genetic confirmation of disease will also be required to replicate, generalize, and extend our findings beyond specialist research settings to the clinic. While our focus in this study was on the most common forms of younger‐onset dementia (i.e., FTD and AD), future studies should explore these profiles in vascular dementia and Lewy body dementia, to provide a truly transdiagnostic overview. More sophisticated models (e.g., event‐based models) and longitudinal designs should also be applied to delineate the relative importance of cognition versus apathy at discrete timepoints in each syndrome, to ensure timely and adaptive care planning.

## 5 CONCLUSIONS

This study highlights the flexibility and utility of GAMs to model trajectories of discrete symptom profiles in younger‐onset dementia syndromes and harness this information to predict functional impairment. Overall, we observed syndrome‐specific trajectories of cognition and apathy, which varied in terms of their linearity and respective impact on functional outcomes. Modeling these dynamic and complex interrelationships affords high prognostic value not only in terms of anticipating potential transitional periods in terms of functional impairment but also highlighting possible windows of opportunity for targeted intervention to better support the person living with younger‐onset dementia and their families.

## CONFLICT OF INTEREST STATEMENT

The authors have no competing interests to declare. No generative AI was used in the writing or preparation of this article.

## ETHICS

Ethics approval for this project was covered under the protocols “Clinical Assessment for Ageing and Neurodegeneration Research” (2020/HE000224) and “Neuroimaging for Ageing and Neurodegeneration Research” (2020/HE000408). This approval was received from the University of Sydney prior to conducting this study and the study was performed in accordance with the ethical standards of the Declaration of Helsinki.

## Supporting information



Supporting Information

Supporting Information

## References

[1] Murley AG , Coyle‐Gilchrist I , Rouse MA , et al. Redefining the multidimensional clinical phenotypes of frontotemporal lobar degeneration syndromes. Brain. 2020;143(5):1555‐1571. doi:10.1093/brain/awaa097 32438414 10.1093/brain/awaa097 PMC7241953

[2] Coyle‐Gilchrist ITS , Dick KM , Patterson K , et al. Prevalence, characteristics, and survival of frontotemporal lobar degeneration syndromes. Neurology. 2016;86(18):1736‐1743. doi:10.1212/WNL.0000000000002638 27037234 10.1212/WNL.0000000000002638 PMC4854589

[3] Ramanan S , El‐Omar H , Roquet D , et al. Mapping behavioural, cognitive and affective transdiagnostic dimensions in frontotemporal dementia. Brain Commun. 2023;5(1):fcac344. doi:10.1093/braincomms/fcac344 36687395 10.1093/braincomms/fcac344 PMC9847565

[4] Vijverberg EGB , Dols A , Krudop WA , et al. Diagnostic accuracy of the frontotemporal dementia consensus criteria in the late‐onset frontal lobe syndrome. Dement Geriatr Cogn Disord. 2016;41(3‐4):210‐219. doi:10.1159/000444849 27160162 10.1159/000444849

[5] Giebel C , Silva‐Ribeiro W , Watson J , et al. A systematic review on the evidence of misdiagnosis in dementia and its impact on accessing dementia care. Int J Geriatr Psychiatry. 2024;39(10):e6158. doi:10.1002/gps.6158 39460409 10.1002/gps.6158

[6] Benussi A , Premi E , Gazzina S , et al. Progression of behavioral disturbances and neuropsychiatric symptoms in patients with genetic frontotemporal dementia. JAMA Netw Open. 2021;4(1):e2030194. doi:10.1001/jamanetworkopen.2020.30194 33404617 10.1001/jamanetworkopen.2020.30194 PMC7788468

[7] Cosseddu M , Benussi A , Gazzina S , et al. Progression of behavioural disturbances in frontotemporal dementia: a longitudinal observational study. Eur J Neurol. 2020;27(2):265‐272. doi:10.1111/ene.14071 31448481 10.1111/ene.14071

[8] Foxe D , Irish M , Cheung SC , et al. Longitudinal changes in functional capacity in frontotemporal dementia and Alzheimer's disease. Alzheimers Dement. 2024;16(4):e70028. doi:10.1002/dad2.70028 10.1002/dad2.70028 PMC11567831 39553250

[9] Poos JM , MacDougall A , van den Berg E , et al. Longitudinal cognitive changes in genetic frontotemporal dementia within the GENFI cohort. Neurology. 2022;99(3):e281‐e295. doi:10.1212/WNL.0000000000200384 35483895 10.1212/WNL.0000000000200384 PMC9302936

[10] Staffaroni AM , Ljubenkov PA , Kornak J , et al. Longitudinal multimodal imaging and clinical endpoints for frontotemporal dementia clinical trials. Brain. 2019;142(2):443‐459. doi:10.1093/brain/awy319 30698757 10.1093/brain/awy319 PMC6351779

[11] Kühnel L , Berger AK , Markussen B , Raket LL . Simultaneous modeling of Alzheimer's disease progression via multiple cognitive scales. Stat Med. 2021;40(14):3251‐3266. doi:10.1002/sim.8932 33853199 10.1002/sim.8932

[12] Verlinden VJA , van der Geest JN , de Bruijn RFAG , Hofman A , Koudstaal PJ , Ikram MA . Trajectories of decline in cognition and daily functioning in preclinical dementia. Alzheimers Dement. 2016;12(2):144‐153. doi:10.1016/j.jalz.2015.08.001 26362597 10.1016/j.jalz.2015.08.001

[13] Yuan M , Rong M , Long X , Lian S , Fang Y . Trajectories of cognitive decline in different domains prior to AD onset in persons with mild cognitive impairment. Arch Gerontol Geriatr. 2024;122:105375. doi:10.1016/j.archger.2024.105375 38431989 10.1016/j.archger.2024.105375

[14] Benussi A , Ashton NJ , Karikari TK , et al. Prodromal frontotemporal dementia: clinical features and predictors of progression. Alz Res Therapy. 2021;13(1):188. doi:10.1186/s13195‐021‐00932‐2 10.1186/s13195-021-00932-2 PMC8594126 34782010

[15] Chatzidimitriou E , Ioannidis P , Moraitou D , Konstantinopoulou E , Aretouli E . The cognitive and behavioral correlates of functional status in patients with frontotemporal dementia: a pilot study. Front Hum Neurosci. 2023;17:1087765. doi:10.3389/fnhum.2023.1087765 36923586 10.3389/fnhum.2023.1087765 PMC10009888

[16] Maito MA , Santamaría‐García H , Moguilner S , et al. Classification of Alzheimer's disease and frontotemporal dementia using routine clinical and cognitive measures across multicentric underrepresented samples: a cross sectional observational study. Lancet Reg Health Am. 2023;17. doi:10.1016/j.lana.2022.100387 10.1016/j.lana.2022.100387 PMC9794191 36583137

[17] Mioshi E , Kipps CM , Hodges JR . Activities of daily living in behavioral variant frontotemporal dementia: differences in caregiver and performance‐based assessments. Alzheimer Dis Assoc Disord. 2009;23(1):70. doi:10.1097/WAD.0b013e318182d293 19266701 10.1097/wad.0b013e318182d293

[18] Mulders AJMJ , Zuidema SU , Verhey FR , Koopmans RTCM . Characteristics of institutionalized young onset dementia patients – the BEYOnD study. Int Psychogeriatr. 2014;26(12):1973‐1981. doi:10.1017/S1041610214001859 25295790 10.1017/S1041610214001859

[19] Kumfor F , Zhen A , Hodges JR , Piguet O , Irish M . Apathy in Alzheimer's disease and frontotemporal dementia: distinct clinical profiles and neural correlates. Cortex. 2018;103:350‐359. doi:10.1016/j.cortex.2018.03.019 29704671 10.1016/j.cortex.2018.03.019

[20] Lansdall CJ , Coyle‐Gilchrist ITS , Jones PS , et al. Apathy and impulsivity in frontotemporal lobar degeneration syndromes. Brain. 2017;140(6):1792‐1807. doi:10.1093/brain/awx101 28486594 10.1093/brain/awx101 PMC5868210

[21] Quang H , Wong S , Husain M , et al. Beyond language impairment: profiles of apathy in primary progressive aphasia. Cortex. 2021;139:73‐85. doi:10.1016/j.cortex.2021.02.028 33836304 10.1016/j.cortex.2021.02.028

[22] Shaw SR , El‐Omar H , Roquet D , et al. Uncovering the prevalence and neural substrates of anhedonia in frontotemporal dementia. Brain. 2021;144(5):1551‐1564. doi:10.1093/brain/awab032 33843983 10.1093/brain/awab032

[23] O'Connor CM , Clemson L , Flanagan E , et al. The Relationship between behavioural changes, cognitive symptoms, and functional disability in primary progressive aphasia: a Longitudinal Study. Dement Geriatr Cogn Disord. 2016;42(3‐4):215‐226. doi:10.1159/000449283 27684067 10.1159/000449283

[24] O'Connor CM , Clemson L , Hornberger M , et al. Longitudinal change in everyday function and behavioral symptoms in frontotemporal dementia. Neurol Clin Pract. 2016;6(5):419‐428. doi:10.1212/CPJ.0000000000000264 27847684 10.1212/CPJ.0000000000000264 PMC5100706

[25] Shaw SR , Horne KS , Piguet O , Ahmed RM , Whitton AE , Irish M . Profiles of motivational impairment and their relationship to functional decline in frontotemporal dementia. J Neurol. 2024;271(8):4963‐4971. doi:10.1007/s00415‐024‐12430‐0 38758282 10.1007/s00415-024-12430-0 PMC11319612

[26] Morrow CB , Kamath V , Dickerson BC , et al. Neuropsychiatric symptoms cluster and fluctuate over time in behavioral variant frontotemporal dementia. Psychiatry Clin Neurosci. 2025;79:327‐335. doi:10.1111/pcn.13810 40079430 10.1111/pcn.13810 PMC12133425

[27] Wood SN . Generalized Additive Models: An Introduction with R, Second Edition. 2nd ed. Chapman and Hall/CRC; 2017. doi:10.1201/9781315370279

[28] Mundo AI , Tipton JR , Muldoon TJ . Generalized additive models to analyze nonlinear trends in biomedical longitudinal data using R: beyond repeated measures ANOVA and linear mixed models. Stat Med. 2022;41(21):4266‐4283. doi:10.1002/sim.9505 35796389 10.1002/sim.9505 PMC9844249

[29] Liu‐Seifert H , Siemers E , Price K , et al. Cognitive impairment precedes and predicts functional impairment in mild Alzheimer's disease. J Alzheimers Disease. 2015;47(1):205‐214. doi:10.3233/JAD‐142508 26402769 10.3233/JAD-142508 PMC4923754

[30] Rascovsky K , Hodges JR , Knopman D , et al. Sensitivity of revised diagnostic criteria for the behavioural variant of frontotemporal dementia. Brain. 2011;134(9):2456‐2477. doi:10.1093/brain/awr179 21810890 10.1093/brain/awr179 PMC3170532

[31] Gorno‐Tempini ML , Hillis AE , Weintraub S , et al. Classification of primary progressive aphasia and its variants. Neurology. 2011;76(11):1006‐1014. doi:10.1212/WNL.0b013e31821103e6 21325651 10.1212/WNL.0b013e31821103e6 PMC3059138

[32] McKhann GM , Knopman DS , Chertkow H , et al. The diagnosis of dementia due to Alzheimer's disease: recommendations from the National Institute on Aging‐Alzheimer's Association workgroups on diagnostic guidelines for Alzheimer's disease. Alzheimers Dement. 2011;7(3):263‐269. doi:10.1016/j.jalz.2011.03.005 21514250 10.1016/j.jalz.2011.03.005 PMC3312024

[33] Foxe D , Irish M , Hu A , et al. Longitudinal cognitive and functional changes in primary progressive aphasia. J Neurol. 2021;268(5):1951‐1961. doi:10.1007/s00415‐020‐10382‐9 33417000 10.1007/s00415-020-10382-9

[34] Mioshi E , Hsieh S , Savage S , Hornberger M , Hodges JR . Clinical staging and disease progression in frontotemporal dementia. Neurology. 2010;74(20):1591‐1597. doi:10.1212/WNL.0b013e3181e04070 20479357 10.1212/WNL.0b013e3181e04070

[35] Lima‐Silva TB , Mioshi E , Bahia VS , et al. Disease progression in frontotemporal dementia and Alzheimer disease: the Contribution of Staging Scales. J Geriatr Psychiatry Neurol. 2021;34(5):397‐404. doi:10.1177/0891988720944239 32762416 10.1177/0891988720944239

[36] Hsieh S , Schubert S , Hoon C , Mioshi E , Hodges JR . Validation of the Addenbrooke's Cognitive Examination III in frontotemporal dementia and Alzheimer's disease. Dement Geriatr Cogn Disord. 2013;36(3‐4):242‐250. doi:10.1159/000351671 23949210 10.1159/000351671

[37] So M , Foxe D , Kumfor F , et al. Addenbrooke's cognitive examination III: psychometric characteristics and relations to functional ability in dementia. J Int Neuropsychol Soc. 2018;24(8):854‐863. doi:10.1017/S1355617718000541 30189909 10.1017/S1355617718000541

[38] Wear HJ , Wedderburn CJ , Mioshi E , et al. The Cambridge Behavioural Inventory revised. Dement neuropsychol. 2008;2:102‐107. doi:10.1590/S1980‐57642009DN20200005 29213551 10.1590/S1980-57642009DN20200005 PMC5619578

[39] Azur MJ , Stuart EA , Frangakis C , Leaf PJ . Multiple imputation by chained equations: what is it and how does it work? Int J Methods Psychiatr Res. 2011;20(1):40‐49. doi:10.1002/mpr.329 21499542 10.1002/mpr.329 PMC3074241

[40] Parameswaran V , Koos H , Kalwani N , et al. Drivers of telemedicine in primary care clinics at a large academic medical centre. J Telemed Telecare. 2023;31:777‐787. doi:10.1177/1357633x231219311 38130140 10.1177/1357633X231219311

[41] Foxe D , Irish M , Ramanan S , et al. Longitudinal changes in behaviour, mood and functional capacity in the primary progressive aphasia variants. Eur J Neurosci. 2022;56(9):5601‐5614. doi:10.1111/ejn.15557 34888957 10.1111/ejn.15557

[42] Irish M , Bunk S , Tu S , et al. Preservation of episodic memory in semantic dementia: the importance of regions beyond the medial temporal lobes. Neuropsychologia. 2016;81:50‐60. doi:10.1016/j.neuropsychologia.2015.12.005 26683384 10.1016/j.neuropsychologia.2015.12.005

[43] Tu S , Wong S , Hodges JR , Irish M , Piguet O , Hornberger M . Lost in spatial translation – A novel tool to objectively assess spatial disorientation in Alzheimer's disease and frontotemporal dementia. Cortex. 2015;67:83‐94. doi:10.1016/j.cortex.2015.03.016 25913063 10.1016/j.cortex.2015.03.016

[44] Kamminga J , Kumfor F , Burrell JR , Piguet O , Hodges JR , Irish M . Differentiating between right‐lateralised semantic dementia and behavioural‐variant frontotemporal dementia: an examination of clinical characteristics and emotion processing. J Neurol Neurosurg Psychiatry. 2015;86(10):1082‐1088. doi:10.1136/jnnp‐2014‐309120 25511791 10.1136/jnnp-2014-309120

[45] Strikwerda‐Brown C , Grilli MD , Andrews‐Hanna J , Irish M . “All is not lost”—Rethinking the nature of memory and the self in dementia. Ageing Res Rev. 2019;54:100932. doi:10.1016/j.arr.2019.100932 31238174 10.1016/j.arr.2019.100932 PMC6726574

[46] Zhang X , Irish M , Piguet O , Ahmed RM . Behavioural and cognitive profiles in frontotemporal dementia and Alzheimer's disease: a longitudinal study. J Neurol. 2025;272(4):279. doi:10.1007/s00415‐025‐13025‐z 40116949 10.1007/s00415-025-13025-z PMC11928428

[47] Massimo L , Kales HC , Kolanowski A . State of the science: apathy as a model for investigating behavioral and psychological symptoms in dementia. J Am Geriatr Soc. 2018;66(S1):S4‐S12. doi:10.1111/jgs.15343 29659001 10.1111/jgs.15343 PMC5905718

[48] Irish M , Ahmed RM . Treating apathy in frontotemporal dementia. Lancet Neurology. 2025;24(2):90‐92. doi:10.1016/S1474‐4422(24)00521‐0 39862888 10.1016/S1474-4422(24)00521-0

[49] Coleman KKL , Berry S , Cummings J , et al. Intranasal oxytocin for apathy in people with frontotemporal dementia (FOXY): a multicentre, randomised, double‐blind, placebo‐controlled, adaptive, crossover, phase 2a/2b superiority trial. Lancet Neurology. 2025;24(2):128‐139. doi:10.1016/S1474‐4422(24)00456‐3 39862881 10.1016/S1474-4422(24)00456-3 PMC13557230

[50] Ahmed RM , Piguet O , Mummery CJ , Naismith SL , Irish M . The Holy Grail: highlighting the need for equitable access to dementia treatments and clinical trials. Lancet Reg Health West Pac. 2025;55. doi:10.1016/j.lanwpc.2025.101492 10.1016/j.lanwpc.2025.101492 PMC11849590 39995763

