# Supplementary material for: Modeling contributions of cognition and apathy to functional impairment in younger‐onset dementia
Source: Alzheimers Dement (Amst). 2026 Jan 20;18(1):e70249. doi: 10.1002/dad2.70249 (PMC12819045; doi:10.1002/dad2.70249)
Supplement: Supplementary file 2 — Supporting Information [file DAD2-18-e70249-s002.docx]

**Supplementary**

**Distinguishing FTD from AD**

Our GAM included both parametric group comparisons based on linear group terms and non-parametric predictions stratified by diagnoses using an interaction (interactions are reported in the main text). The linear group comparisons showed that each FTD subgroup was significantly different from AD in terms of functional impairment as measured by the FRS. Specifically, the bvFTD group showed greater functional impairment relative to AD while SD and PNFA were milder (see Supplementary Table 1).

**Supplementary Table 1.** Profiles of functional impairment in FTD subtypes relative to AD

| Groups | Estimate | Std. Error | z-value | p-value |
| --- | --- | --- | --- | --- |
| Intercept (AD) | 1.22 | 0.04 | 32.74 | <.001* |
| bvFTD | -0.23 | 0.06 | -3.84 | <.001* |
| PNFA | 0.22 | 0.07 | 3.07 | .002* |
| SD | 0.13 | 0.06 | 2.20 | .028* |

*Significant at α =.05 level. This model compares disease severity scores (i.e., functional impairment) on the FRS when controlling for age and education. The estimate score is the difference in a group’s mean FRS score in reference to the AD group, where lower scores reflect greater functional impairment. Note: The FRS was shifted by 3.09 (i.e., the lowest negative value) so that all values were positive to facilitate a scaled t-family GAM with a log function (see statistical analysis section for further details).

**Examining sex effects**

We examined if the original model would differ when sex was added as a covariate. However, no major differences were observed at the model or coefficient level (See Supplementary Tables 2 and 3).

**Supplementary Table 2.** Parametric model results when covarying for sex

| Groups | Estimate | Std. Error | z-value | p-value |
| --- | --- | --- | --- | --- |
| Intercept (AD) | 1.24 | 0.04 | 32.05 | <.001* |
| bvFTD | -0.23 | 0.06 | -3.79 | <.001* |
| PNFA | 0.22 | 0.07 | 3.15 | .002* |
| SD | 0.14 | 0.06 | 2.35 | .019* |
| Sex (Female) | -0.04 | 0.03 | -1.37 | .171 |

*Significant at α =.05 level. This model compares disease severity scores (i.e., functional impairment) on the FRS when controlling for age, sex, and education. The estimate score is the difference in a group’s mean FRS score in reference to the AD group, where lower scores reflect greater functional impairment. Note: The FRS was shifted by 3.09 (i.e., the lowest negative value) so that all values were positive to facilitate a scaled t-family GAM with a log function (see statistical analysis section for further details).

**Supplementary Table 3. GAM predictions when covarying for sex**

| **Domain** | **Predictors** | **edf** | **Ref.df** | **Chi.sq** | **p-value** |
| --- | --- | --- | --- | --- | --- |
| Demographic covariates | Age | 1.00 | 1.00 | 0.26 | .610 |
|  | Education | 1.00 | 1.00 | 2.52 | .113 |
| Cognition  (ACE-III) | AD | 1.00 | 1.00 | 27.68 | <.001* |
|  | bvFTD | 1.00 | 1.00 | 21.92 | <.001* |
|  | PNFA | 1.00 | 1.00 | 15.84 | <.001* |
|  | SD | 3.26 | 4.05 | 10.55 | .034* |
| Apathy  (CBI-Apathy) | AD | 1.00 | 1.00 | 52.23 | <.001* |
|  | bvFTD | 1.69 | 2.11 | 39.80 | <.001* |
|  | PNFA | 2.14 | 2.66 | 22.45 | <.001* |
|  | SD | 2.37 | 2.96 | 68.47 | <.001* |

Note: Apathy was measured using the Cambridge Behavioural Inventory Revised (CBI-R) motivation subscale, cognition using the Addenbrooke's Cognitive Examination III (ACE-III) total score, and functional impairment using the Frontotemporal Dementia Rating Scale (FRS) logit score. AD = Alzheimer’s disease, SD = semantic dementia, PNFA = progressive nonfluent aphasia, bvFTD = behavioural-variant frontotemporal dementia.

* Significant at α =.05 level.
